# How culture and environment reshape the adoption and effectiveness of wearable diet monitoring in university students: an extended KANO–TAM–SEM model study using Taiwan as an example

**DOI:** 10.3389/fpsyg.2026.1795601

**Published:** 2026-04-07

**Authors:** Chen Han, Chuanchia Wang, Jwoshiun Sun, Tungjing Fang

**Affiliations:** 1 College of Design, National Taipei University of Technology, Taipei, Taiwan; 2 Department of Electronic Engineering, National Taipei University of Technology, Taipei, Taiwan; 3 Institute of Physiology and School of Medicine, National Defense Medical University, Taipei, Taiwan

**Keywords:** culture, dietary self-regulation, environment, Kano model, structural equation modeling, Taiwan university students, technology acceptance model, wearable technology

## Abstract

**Background:**

A healthy lifestyle contributes to optimal nutrition and physical well-being. However, the cognitive and feedback regulation of hunger at psychophysiological levels—as a core mechanism for food intake control and self-regulation—remains understudied. University students commonly exhibit a gap between nutritional knowledge and practice. In Taiwan, the prevalence of lifestyle-related chronic diseases coexists with universal health insurance coverage, highlighting the urgent need for culturally adapted, user-centered technological interventions to enhance daily self-management capabilities.

**Objective:**

Guided by the KANO model, this study integrates Culture, Environment, Habit, Self-Perception, and User Evaluation (CEHSV) into a unified KANO-TAM-SEM framework, extending the Technology Acceptance Model (TAM) to explain adoption behaviors and downstream outcomes of dietary tracking wearables.

**Methods:**

The present study employed a single-arm, prospective field intervention with pre–post assessments (within-subject longitudinal design). During a 12-week field intervention, N = 200 university students (mean age 20.4 ± 1.8 years; 62% female) used a human-centered application designed based on the Kano model (Basic Functions: Accuracy/Reliability/Simplicity; Efficacy features: goal setting and feedback; Attractiveness features: gamification and social sharing). Evaluation metrics included: fruit and vegetable intake portions, dietary self-regulation scores, daily step counts, and WHOQOL-BREF quality-of-life scales. Structural equation modeling (SEM) validated the theoretical framework; multi-group SEM separately examined dormitory vs. home living environments and self-efficacy subgroups.

**Results:**

93% completion rate; food recording coverage ~78% (5.5 days/week). Fruit and vegetable intake increased by +1.1 servings/day; The results of the study demonstrated a significant increase in dietary self-regulation scores, from 64.59 to 79.50, on a scale ranging from 0 to 100 (*Δ* = +16.17, paired test *p* < 0.001); The WHOQOL-BREF overall score increased from 70.75 to 77.23 (Δ = +6.59; p < 0.001); BMI (kg/m^2^) was incorporated into the analysis. The data were reported with the mean ± standard deviation (SD), the delta (*Δ*), the paired t-test, and the effect size. BMI remained stable (22.4 at both pre- and post-measurement). Model fit was excellent (χ^2^/df = 1.87, CFI = 0.98, RMSEA = 0.032), with all hypothesized paths supported (*p* < 0.01). The extended model explained 36% of usage frequency, 45% of dietary self-regulation improvement, and 54% of quality-of-life enhancement, significantly surpassing baseline effects observed with TAM alone (approximately 7% usage rate; ~39% dietary change). This model structure was validated across dormitory/home environments and across low- and high-self-efficacy groups.

**Conclusion:**

Integrating CEHSV contextual factors into TAM establishes a culturally intelligent and user-centered framework for preventive dietary wearables. This framework bridges the knowledge-practice gap, thereby enhancing health and well-being.

## 1 Introduction

University years represent a critical period for the development of dietary behavior. However, when academic pressure, time constraints, and social activities collide, students often fall into a vicious cycle of irregular meals, high intake of processed foods, and late-night eating. The gap between nutritional knowledge and dietary practice is well documented, indicating that mere information dissemination is insufficient—students often understand healthy eating principles but struggle to put them into practice (Phelan et al., 2025; Parks et al., 2025; Oh, 2025; Almoraie et al., 2025). This cognition-practice gap necessitates innovative approaches to transform health knowledge into enduring healthy habits.

Nutrition is a vital component of human life, playing a crucial role in maintaining health and well-being. However, the nutritional status of college students has drawn significant attention in recent years (Suárez-Reyes et al., 2019). As young people transition from adolescence to young adulthood, they face numerous challenges related to healthy dietary choices (Stok et al., 2018). This period also gives rise to new behavioral patterns (Sogari et al., 2018), cases of poor dietary habits and weight gain rise concurrently (Papadaki et al., 2007). Many students struggle to maintain a healthy diet due to factors such as demanding schedules, stress, limited access to nutritious foods, and poor dietary habits (Escoto et al., 2012; Almoraie et al., 2021).

A healthy lifestyle serves as the cornerstone of achieving optimal nutrition and physical health. However, the psychophysiological recognition and feedback regulation of the hungry stomach, which are paramount for effectively controlling food intake and supporting optimal self-regulation and mental health, remain largely unexplored. Over the past century, psychological well-being—encompassing positive emotions, life satisfaction, and favorable psychological and social functioning—has emerged as a crucial component of mental health, reflecting a positive state of health distinct from negative mental health disorders (Keyes, 2006; Keyes et al., 2008; Westerhof and CLM, 2009; Seligman and Csikszentmihalyi, 2000). Psychological well-being correlates with multiple positive outcomes, including resilience (Fredrickson, 2001; DuPont et al., 2020; Tugade, 2010; Tugade and Fredrickson, 2004), enhanced physical health and longevity (Charles and Carstensen, 2010; Danner et al., 2001; Diener et al., 2017; Howell et al., 2007; Pressman and Cohen, 2005; Pressman et al., 2019; Reinilä et al., 2023; Steptoe et al., 2015), and workplace creativity (Acar et al., 2020; Baas et al., 2008; Conner et al., 2016; Davis, 2009; Graziotin et al., 2014). Furthermore, young people face multiple challenges in maintaining healthy eating habits: a lack of interest among males, the influence of poor dietary habits among peers and family members, the affordability of unhealthy foods, time constraints, inadequate cooking facilities, and insufficient nutrition knowledge. These compounding challenges significantly hinder young people’s ability to establish and maintain nutritious eating habits (Munt et al., 2017). Young adults (aged 17–25) (National Research Council Institute of Medicine, 2015; Conley et al., 2020; Wickham et al., 2020). Obesity rates among university students have risen significantly in recent years, increasing the risk of chronic diseases such as diabetes and heart disease (Papadaki et al., 2007; Almoraie et al., 2021). Furthermore, nutritionally inadequate diets may trigger mental health issues, including depression and anxiety, and lead to declining academic performance (Langford et al., 2014). According to multiple studies on university students, the prevalence of overweight and obesity is approximately 30%, with male students exhibiting higher rates than female students (Peltzer et al., 2014). Mental health disorders among youth exhibit the highest prevalence rates, reaching up to 22% (National Institute of Mental Health, 2016). Research also indicates that many students’ diets lack essential nutrients such as vitamins and minerals. Many students tend to consume high-calorie, high-sugar, and high-fat foods, including fast food, processed snacks, and sugary beverages (Almoraie et al., 2021; Langford et al., 2014). Unhealthy diets may trigger various health issues, including fatigue, poor concentration, and weakened immune systems (Almoraie et al., 2021; Althunibat et al., 2023). The scarcity of nutritious food options on campus exacerbates this problem (Papadaki et al., 2007; Larson et al., 2009; Almoraie et al., 2021; Langford et al., 2014), potentially leading to insufficient energy and nutritional deficiencies that impair academic performance (Merhy et al., 2023). Furthermore, students often consume high-calorie foods like pizza, potato chips, and ice cream while studying late into the night. This can result in weight gain and reduced sleep quality due to late-night snacking (Alafif and Alruwaili, 2023). This particular vulnerability underscores the importance of researching how to promote a healthy diet and well-being effectively.

Wearable technology and mobile health (mHealth) systems can help bridge this gap between intention and behavior by providing continuous self-monitoring, real-time reminders, and feedback loops that support goal setting and reflective regulation. However, system adoption rates and sustained engagement represent critical bottlenecks. If users fail to engage with the system frequently enough to form behavioral learning, the system cannot improve dietary behaviors. Existing research often employs frameworks such as the Technology Acceptance Model (TAM) to model adoption behavior. This model posits that perceived ease of use (PEOU) and perceived usefulness (PU) jointly shape both the intention to use and actual usage behavior (Davis, 1989). Although the TAM model retains a solid foundation (Ajzen, 1991), its explanation of how context shapes acceptance and outcomes remains insufficient—particularly for behaviors such as eating, which are socially and culturally contextualized. Health behavior technology differs from health information technology in that its use is voluntary, its effects emerge gradually, and social recognition and cultural norms influence whether users adopt or disregard feedback.

Taiwan offers an illuminating macro-context: while healthcare accessibility is exceptionally high (nearly universal health insurance coverage), lifestyle-related risks remain significant (Cheng, 2015; Lu and Hsiao, 2003). Taiwan’s NHI has achieved near-universal coverage (99.6%), yet the prevalence of lifestyle-related chronic diseases remains significant (National Health Insurance Administration, 2017). For instance, the prevalence of treated diabetes reached 11.2% in 2020 (>2.58 million cases), and nationally representative NAHSIT-based analyses have documented the sustained obesity and rising abdominal obesity burden over time (National Health Insurance Administration, 2017; Nutrition and Health Survey in Taiwan (NAHSIT), 2025). This paradox reveals a prevention gap—health outcomes depend more on daily self-regulation than clinical resources. Against this backdrop, wearable dietary systems are not merely products but socio-technical interventions requiring alignment with local culture, living environments, and behavioral patterns to be effective.

Universities can play a pivotal role in promoting healthy eating habits by providing nutritious food options on campus and advocating for the importance of good nutrition (Abraham et al., 2018; Hafiz et al., 2023). One effective strategy to target college students’ dietary behaviors is to implement nutrition education programs. Although designing tailored nutrition education programs for young populations presents challenges, such initiatives are crucial for public health (Moschonis et al., 2021). However, further research is needed to determine optimal implementation methods (Chau et al., 2018; Hamulka et al., 2018).

This study proposes a context-aware extension model based on the KANO-TAM-SEM framework, incorporating five human-centered determinants beyond classic acceptance factors: culture (health orientation and externalized medical dependency), environment (living situations and food access channels), habits (behavioral routines and automation risks), self-perception (dietary self-efficacy and change willingness), and user evaluation (usability and satisfaction). These “context-health-self-efficacy-values” constructs are integrated into a unified structural model linking technology adoption to dietary self-regulation and quality-of-life outcomes. The Kano product attribute model complements TAM by ensuring the system satisfies basic expectations (must-have features), performance requirements, and surprise factors (Kano et al., 1984). Building upon this foundation, the CEHSV extension model in this study positions situational and personal factors as antecedents to TAM cognition and usage behavior.

**Figure 1:**
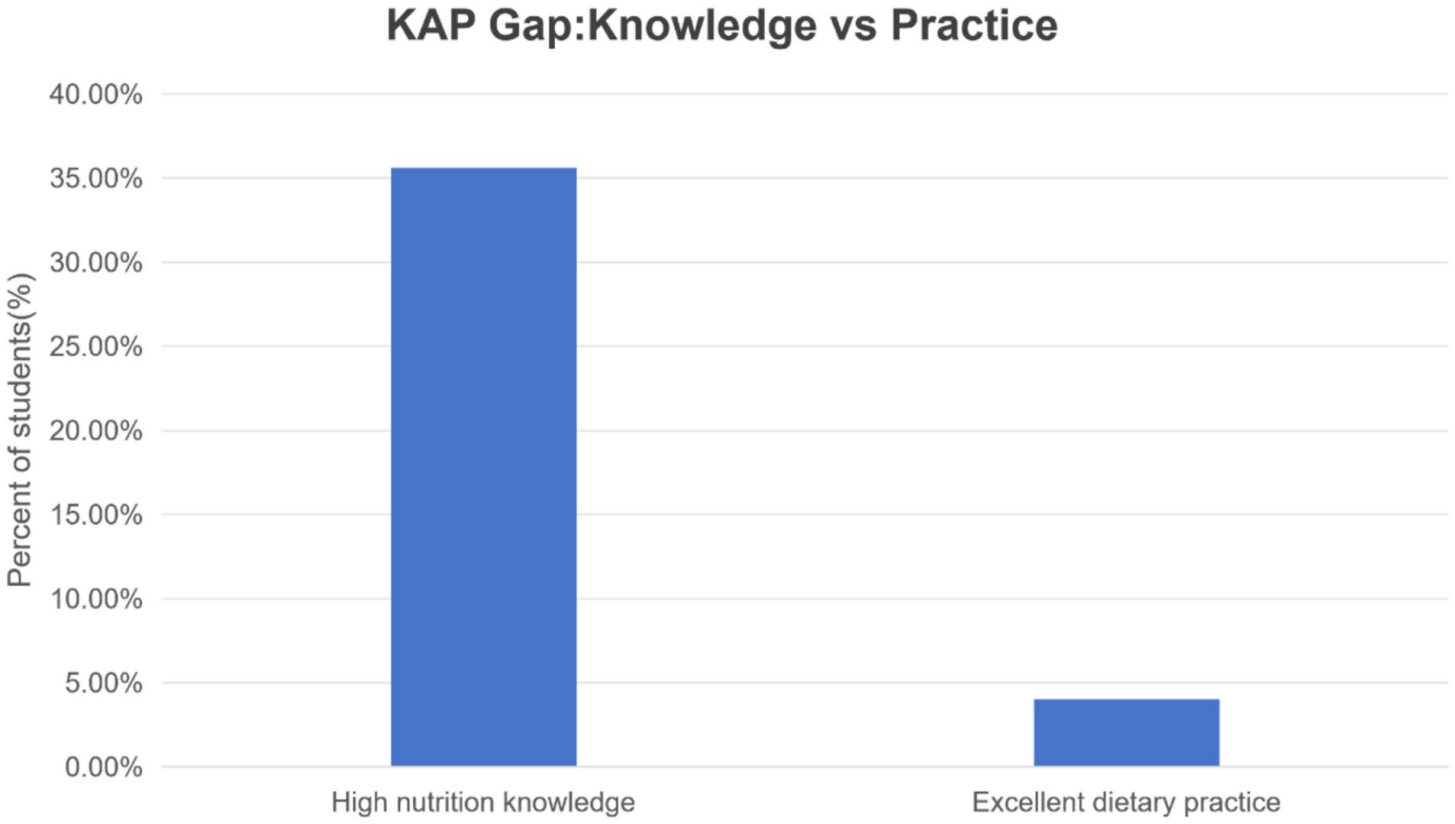
Knowledge–practice gap drives context-aware interventions. A simple comparison illustrates this gap: most students possess solid nutritional knowledge, yet only a minority maintain excellent dietary habits. This discrepancy—where knowledge far exceeds behavior—highlights the inadequacies of traditional education and compels us to focus on interventions that provide sustained support for self-regulation.

As illustrated in Figure 1, a conceptual schematic of the knowledge-practice gap motivating context-aware interventions is presented. It is important to note that the displayed values are illustrative and not derived from the present sample.

The primary model under scrutiny examines indirect (mediated) pathways from contextual antecedents through TAM cognitions to usage and subsequent outcomes. Multi-group SEM analyses evaluate whether selected structural paths differ across living environment or self-efficacy strata (moderation/heterogeneity). This model posits that these contextual factors will influence perceived ease of use (PEOU) and perceived usefulness (PU), subsequently affect engagement (usage rate), and ultimately drive changes in dietary self-regulation behavior (*Δ* Dietary Behavior) and quality of life (Δ Quality of Life).

This study is positioned as original research. Through empirical field investigations among Taiwanese university students, it employs multi-group structural equation modeling (SEM) to examine the heterogeneity generated by environmental settings (dormitory vs. home) and self-perception types (low vs. high dietary self-efficacy).

## 2 Related work

While findings on the impact of wearable devices and mobile health interventions on student dietary and lifestyle behaviors are mixed, the potential remains significant. Most programs rely on self-monitoring, goal setting, and feedback mechanisms—behavior change techniques known to enhance self-regulation (Ferguson et al., 2022). High dropout rates in digital health trials underscore the critical importance of design elements that sustain motivation (Khoury et al., 2016).

Technology Acceptance Modeling (TAM) provides a systematic perspective for understanding early adoption behaviors. The TAM concepts of “perceived usefulness” and “perceived ease of use” have been extended in health contexts to include factors like trust and outcome expectancy (Venkatesh et al., 2012). However, the classic TAM does not explicitly incorporate cultural beliefs or habit strength. In the health behavior domain, the Theory of Planned Behavior (Ajzen, 1991) indicates that subjective norms (culture) and perceived control (self-efficacy) influence intention and behavior, yet technology-oriented models often overlook these constructs.

The Kano model offers a complementary design perspective (Kano et al., 1984). This model categorizes product features into three types: Must-Have (basic quality expected by users to prevent dissatisfaction), Performance (linear satisfaction growth with enhanced functionality), and Attraction (unexpected delight that significantly boosts satisfaction when present, without causing dissatisfaction when absent). For behavior change tools, essential features may include reliability and low friction (to prevent attrition due to frustration); performance features could use clear feedback and progress tracking (to increase user satisfaction in proportion); and appeal features might use gamification or social comparison (to provide motivation and bursts of enjoyment). Early implementation of appeal features can mitigate mid-process engagement decline—Table 1 lists this system’s features categorized according to the Kano model.

**Table 1:** Baseline characteristics.

Variable	Summary	Value
Age (years)	Mean ± SD	20.4 ± 1.8
Sex	Female, *n* (%)	124 (62.0%)
Sex	Male, *n* (%)	76 (38.0%)
Accommodation (1)	Dormitory, *n* (%)	107 (53.5%)
Accommodation (2)	Home/off-campus with family, *n* (%)	93 (46.5%)
BMI (kg/m^2^)	Mean ± SD	22.4 ± 3.1
Fruit & vegetable servings/day	Mean ± SD	3.2 ± 2.2
Steps/day (wearable)	Mean ± SD	6275.57 ± 1844
Prior nutrition/health app use	Yes, n (%)	16 (8%)

Table 2 presents key research variables and their measurement characteristics. We define “culture” as overt health orientation (5 items, 1–5 scale; e.g., “When I am sick, I tend to rely on doctors/medication rather than changing my habits”), environment as living situation (dormitory vs. family residence, dichotomous classification); habits as an indicator of unhealthy eating patterns (5 behavioral items scored 1–5); self-perception as dietary self-efficacy (8 items, 1–5 scale; e.g., “I can maintain healthy eating even when under stress”). Additionally, standard TAM constructs—Perceived Efficacy of Use (PEOU, 4 items, 1–7 scale) and Perceived Usefulness (PU, 5 items, 1–7 scale)—were adapted for dietary apps (Davis, 1989). User evaluation combined the post-study System Usability Scale (0–100) and satisfaction rating (1–7). Engagement was measured by actual usage rate (proportion of active days, 0–1). Outcome measures included changes in dietary self-regulation (improvement in the Healthful Eating Behavior Composite Score, 0–100) and quality of life (baseline-to-follow-up difference in the WHOQOL-BQ score, 0–100).

**Table 2:** Research constructs, scales, and example items.

Construct	Items	Scale	Example item/indicator
Culture (externalized health orientation)	5	1–5	I rely on doctors/medicine rather than daily self-management
Environment (living context)	1	binary	Dormitory vs. living with family
Habit (unhealthy eating routine)	5	1–5	Late-night snacking, sugary drinks, breakfast skipping, etc.
Self-perception (dietary self-efficacy)	8	1–5	I can maintain healthy eating even when stressed
PEOU	4	1–7	Learning to use the system is easy for me
PU	5	1–7	Using the system helps me eat healthier
User Evaluation (SUS + satisfaction)	11	0–100/1–7	Overall usability and satisfaction
Engagement (usage rate)	—	0–1	% of days active use during 12 weeks
Dietary self-regulation outcome	20	0–100	Meal planning, mindful eating, balanced intake
Quality of life (WHOQOL-BREF)	26	0–100	Physical, psychological, social, and environmental well-being

Summarizes baseline and outcome measurement indicators, including item counts and scale ranges. Each construct is illustrated with example items to facilitate interpretation and replication.

### 2.1 Hypotheses

**Figure 2:**
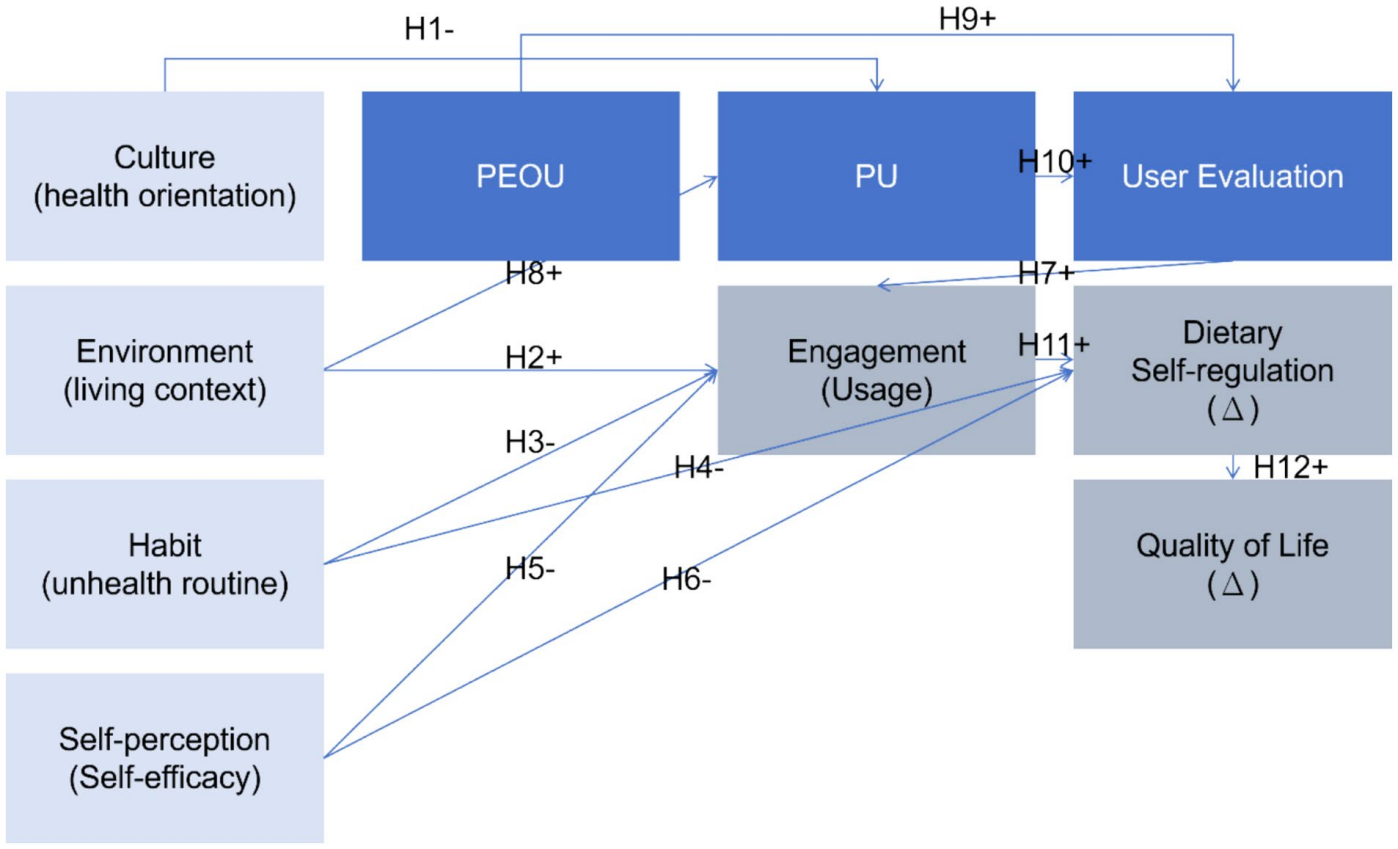
CEHSV extended KANO–TAM–SEM (conceptual model). This model integrates cultural, environmental, habitual, self-perception, and user-evaluation factors to form an integrated framework alongside TAM’s core constructs. Hypotheses H1–H12 (solid arrows) represent predicted pathways.

Based on the literature and our situational model, we propose the following hypotheses (H1–H12 correspond to path labels in Figure 2; H13–H14 explore anticipated group differences):

*H1* (Culture → Perceived Utility): A more externalized health orientation (attributing responsibility to physicians rather than oneself) negatively impacts the perceived utility of dietary self-monitoring tools. Students who believe “health is the doctor’s responsibility” will perceive self-monitoring devices as less beneficial to them.

*H2* (Environment → Usage): Compared to home environments, dormitory living (a more independent setting) positively influences engagement/usage frequency. Dormitory students may require more self-monitoring, leading to more frequent use of the tool.

*H3* (Habit → Usage): The more entrenched unhealthy eating habits are, the more they reduce engagement/usage rates. Deep-rooted bad habits may make dietary tracking tools seem ineffective or more burdensome.

*H4* (Habit → Dietary Change): The stronger unhealthy habits are, the less conducive they are to dietary improvement. Individuals with poorer baseline habits show fewer minor improvements in dietary self-regulation, as ingrained habits hinder behavioral change.

*H5* (Self-Perception → Usage Frequency): Higher dietary self-efficacy (confidence in controlling one’s diet) positively influences engagement/usage frequency. Students who believe they can manage their diet are more likely to use support tools actively.

*H6* (Self-Perception → Dietary Change): Higher dietary self-efficacy positively impacts dietary improvement. Confidence helps translate intentions into action.

*H7* (User Evaluation → Usage): More positive user evaluations (better usability and satisfaction) positively influence engagement/usage rates. Students who find the app enjoyable and valuable will use it more consistently.

*H8* (Perceived Usability → Perceived Usefulness): Perceived usability positively influences perceived usefulness (a classic TAM relationship). An easy-to-operate app is more likely to be perceived as an effective tool for achieving health goals.

*H9* (Perceived Usability → User Evaluation): Usability positively influences user evaluation (satisfaction). A clean, intuitive interface enhances overall satisfaction.

*H10* (Perceived Usefulness → User Evaluation): Perceived usefulness positively influences user evaluation. If students perceive wearable devices as aiding healthy eating, they will be more satisfied with them.

*H11* (Usage Frequency → Dietary Change): Higher engagement/usage frequency will promote improved dietary self-regulation. Consistent tool use should facilitate behavioral change.

*H12* (Dietary Change → Quality of Life Change): An improved diet will enhance quality of life. Better eating habits can translate into improved physical and mental well-being.

*H13* (Multi-group – Environment): Structural pathways may vary by living environment (dormitory vs. home). We anticipate some path coefficients will differ across groups (e.g., the influence of habits or user satisfaction on usage frequency may be more pronounced in specific environments). However, we also hypothesize that core pathways (usage → outcomes) remain consistent across environments.

*H14* (Multi-group – Self-perception): Participation drivers may vary by self-efficacy levels. For instance, users with low self-efficacy may rely more on external cues (environmental or app features) to engage; conversely, those with high self-efficacy may participate without external prompts or be driven more by intrinsic satisfaction.

These hypotheses reflect our expectation that integrating contextual factors (culture, environment, habits, self-perception) with the Technology Acceptance Model (TAM) will provide a more comprehensive explanation of who will adopt the technology and who will benefit from it. Table 3 in the subsequent results section will summarize whether the data support each hypothesis.

**Table 3:** Kano classification of core product features.

Feature	Kano category
Accurate step count	Must-be
Reliable syncing	Must-be
Low battery drain	Must-be
Quick meal logging	Performance
Personalized goals	Performance
Nutrition balance feedback	Performance
Badges & streaks	Attractive
Peer challenges	Attractive
Recipe sharing	Attractive
Clinician-endorsed tips	Attractive

## 3 Method

Study design and context: the present study employed a single-arm, prospective field intervention with pre- and post–assessments (within-subject longitudinal design). The single-arm field design was selected to evaluate real-world adoption and mechanism testing under ecological conditions; findings should be interpreted as associations consistent with the intervention period rather than causal effects. We conducted a 12-week prospective field intervention study at a large university in northern Taiwan. This study employed an original experimental design to evaluate behavioral outcomes and mechanisms of technology adoption. Participants were assessed at baseline (pre-intervention) and 12 weeks post-intervention (post-intervention) to measure changes.

Participants: Two hundred undergraduate students (aged 18–30 years, mean 20.4 ± 1.8 years; 62% female) were recruited via campus email lists and posters. As a key grouping variable, each student’s living environment (on-campus residence hall vs. off-campus residence with family) was recorded. Results showed roughly equal distribution: 107 dormitory residents and 93 family-living residents. All participants provided informed consent, and the study protocol was approved by the university’s Institutional Review Board (IRB No. KY2021-609).

Table 1 presents a comprehensive overview of the study participants’ demographic information, including their age, gender, and accommodation status. It also provides essential health metrics, including their baseline BMI, habitual intake of fruits and vegetables, and daily step count. Additionally, the table offers insights into their prior experience with health applications and wearable technology.

Intervention: each participant received a wearable device and installed a companion smartphone application. The application was developed following human-centered design principles and optimized based on functional Kanban analysis. We prioritized essential features while incorporating several performance features (e.g., clear nutritional feedback and personalized goal setting). Table 3 outlines core features and their Kano classification.

This wearable system’s features are categorized by Kano attributes: Essential features (e.g., precise step counting, stable synchronization, low power consumption) must function flawlessly to prevent user frustration. Performance features (e.g., quick meal logging, personalized goals, nutritional balance feedback) directly enhance user satisfaction, with effectiveness proportional to their quality. Attraction features (e.g., badges and streak records, peer challenges, recipe sharing, and clinically certified expert advice) deliver delightful experiences that extend user engagement.

**Figure 3:**
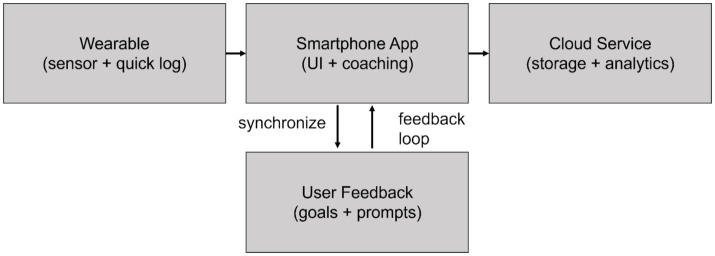
Companion app interface and wearable device system data flow.

During the 12-week trial, students were instructed to use the app to log dietary intake and physical activity. This design, combining sensor data with self-reported entries, aimed to enable continuous self-regulation through a feedback loop: Data → Insights → Behavioral Adjustment. Figure 3 illustrates the app interface and data flow.

The core app screen (schematic) includes the main dashboard, the quick meal logging interface, and the feedback summary screen. Design emphasizes low-friction input and clear feedback. (B) The system architecture diagram illustrates the interaction model among wearable devices, mobile applications, and cloud services: wearable devices capture data, mobile applications analyze it, and provide feedback to users (e.g., goal-setting prompts). Together, these three components depict the user-device interaction loop, and continuously sensed data is transformed into actionable feedback supporting self-regulation (Table 4).

**Table 4:** Pre- and post-intervention comparisons of health outcomes.

Outcome	Baseline mean	Follow-up mean	Change
Diet self-regulation score (0–100)	64.59	79.50	16.17
Fruit & vegetable servings/day	3.2	4.3	1.1
Steps/day	6275.57	8337.96	2062.39
WHOQOL overall (0–100)	70.75	77.23	6.59
Usage rate (% days)	nan	48.50	nan
SUS (0–100)	nan	71.52	nan

Baseline and follow-up means for key indicators, along with absolute changes over 12 weeks. Dietary self-regulation scores improved by approximately 15 points (on a 0–100 scale), fruit and vegetable intake increased by 1.1 servings/day, daily step count rose by about 2,060 steps, and WHOQOL overall scores increased by approximately 6.5 points. Body Mass Index remained broadly stable (not shown), indicating that changes during this phase primarily manifested in behavioral and perceptual dimensions (quality of life) rather than weight shifts.

**Figure 4:**
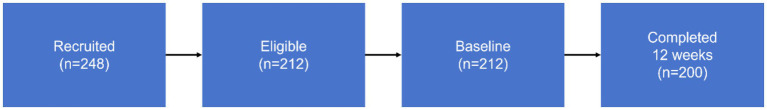
Study participant recruitment and intervention flowchart.

The recruitment and retention overview is shown in Figure 4. Among 248 initially screened students, 212 met eligibility criteria (e.g., no medical dietary restrictions) and completed baseline surveys; 200 completed the full 12-week program (93% retention rate, with 12 withdrawals due to personal reasons or loss to follow-up). This high completion rate reflects strong engagement, potentially attributable to appealing design elements and the relatively brief study duration.

Measurement metrics: multiple self-reported questionnaires and device data were collected:

- Cultural orientation: five scales (custom-developed for this study) measured cultural health orientation at baseline, focusing on internalized vs. externalized tendencies regarding health control (e.g., reliance on physicians vs. self-care). Higher scores indicate stronger external orientation (greater dependence on external medical solutions).
- Habit intensity: assessed baseline dietary habits through five behavioral frequency measures (skipping breakfast, late-night eating, sugary drinks, fast food consumption, lack of home-cooked meals). Standardized and combined into an “Unhealthy Habit Index” (higher values indicate more vigorous unhealthy habit intensity).
- Self-perception (dietary self-efficacy): an 8-item scale adapted from the Dietary Self-Efficacy Questionnaire (1 = strongly disagree, 5 = strongly agree). This scale measures participants’ confidence in various challenging scenarios (e.g., “I can avoid unhealthy snacks even under pressure”). Higher scores indicate greater confidence in maintaining healthy eating habits. The Diet Self-Regulation (DSE) score was calculated as the mean of standardized components, including z (FV servings/day), z (reverse unhealthy snack frequency), and z (FFQ healthy diet index). These components were linearly rescaled to 0–100.
- Perceived ease of use (PEOU): after 12 weeks of system use, participants rated four aspects of the app’s learning and usability (1–7 Likert scale). This scale adapts tools from Davis, 1989 Technology Acceptance Model.
- Perceived usefulness (PU): also assessed post-intervention, this involved five ratings (1–7 points) evaluating the system’s effectiveness in promoting healthy eating, enhancing dietary awareness, and achieving goals.
- User evaluation: assessed using the System Usability Scale (SUS), a standardized usability questionnaire comprising 10 items (0–100 points), along with overall satisfaction (1–7 points) and recommendation intent (1–7 points). These were normalized and combined into a composite user evaluation score. The user evaluation is calculated as follows:
SUS+100×(Satisfaction−1)/6+100×(Recommendation−1)/6/3
- Engagement: the application automatically logs usage data. “Usage rate” is defined as the percentage of days within the 84-day (12-week) period when participants actively recorded at least one meal or viewed feedback. For example, a student participating for 65 of 84 days achieves a usage rate of 77.4%. Daily step counts tracked via wearable devices serve as a secondary engagement metric.
- Dietary Self-Regulation Effectiveness: We calculated a composite healthy eating score at baseline and follow-up, incorporating self-reported fruit/vegetable portion sizes, unhealthy snack frequency, and the Brief Food Frequency Questionnaire, scaled from 0 to 100. The change value (follow-up minus baseline) represents “*Δ* Dietary Self-Regulation,” where positive change indicates improvement.
- Physical activity: we compared average daily step counts (from wearable device data) between the first and last weeks, calculating the overall change in daily steps.
- Quality of life: we used the World Health Organization Quality of Life Brief Version (WHOQOL-BREF) (World Health Organization, 1998), which generates an overall score ranging from 0 to 100. Δ Quality of life represents the change from baseline to follow-up.

The culture (externalized health orientation) scale was evaluated using EFA (1-factor solution; loadings reported) and CFA (standardized loadings, fit indices). The reliability of these estimates is supported by internal consistency (Cronbach’s *α*) and convergent validity (CR, AVE) metrics documented in the supplementary material. CMV was assessed using Harman’s single-factor test and by comparing a single-factor confirmatory factor analysis (CFA) against the theorized measurement model.

- EFA: KMO, Bartlett’s test, extraction (PA/ML), rotation (none for 1-factor), variance explained, loading range.
- CFA: χ2, df, CFI, RMSEA, SRMR; standardized loadings; residual checks.
- Reliability: Cronbach’s α; Composite Reliability (CR); Average Variance Extracted (AVE).
- Dimensionality justification: why 1-factor (or multi-factor) is retained.

CR and AVE formulas (standardized solution):

Given standardized loadings λᵢ and error variances θᵢ:

$$\mathrm{CR}=\frac{{\left(\sum \lambda_{i}\right)}^{2}}{{\left(\sum \lambda_{i}\right)}^{2}+\sum \theta_{i}}\;\;\;\;\mathrm{AVE}=\frac{\sum \lambda_{i}^{2}}{\sum \lambda_{i}^{2}+\sum \theta_{i}}$$

The present study fitted a nested TAM-only baseline structural equation model (SEM) and compared it against the extended CEHSV model using a χ^2^ difference test and changes in explained variance (R^2^). The number of freely estimated parameters in the measurement structural model is reported, and the rationale for *N* = 200 is justified with reference to SEM sample size lower-bound considerations that depend on model complexity and indicator-to-latent ratios. The extended model provided a better fit and higher explained variance than the TAM-only baseline.

Table 2 summarizes these constructs, including scales and example items for reference. All multi-item scales demonstrated good internal consistency (Cronbach’s alpha coefficients ranging from 0.79 to 0.94; reliability metrics detailed in the Results section). Prior to hypothesis testing, we validated the measurement model. All factor loadings were statistically significant (*p* < 0.001), composite reliability (CR) for each construct exceeded 0.8, and average variance extracted (AVE) reached or exceeded the 0.5 threshold.

Statistical Analysis: We constructed a structural equation model reflecting the expanded CEHSV framework (Figure 2). Model estimation employed maximum likelihood in AMOS v26 for structural equation modeling, cross-validated with the lavaan package in R. A baseline model containing only the technological acceptance model relationships (PEOU, PU, user evaluation, usage behavior, outcomes) was first established as a reference. Subsequently, the extended model, incorporating association paths among cultural, environmental, habitual, and self-efficacy variables, was fitted. Model fit was assessed using the chi-square/degrees of freedom ratio (χ^2^/df), comparative fit index (CFI), root mean square error of approximation (RMSEA), and standardized residual ratio (RMR). We considered CFI ≥ 0.95 and RMSEA ≤ 0.06 as indicators of good fit (Cheung and Rensvold, 2002).

For hypothesis testing, we examined the significance (*p*-values) and standardized coefficients (*β*) of each path. Additionally, for key endogenous variables (usage behavior, dietary changes, quality-of-life changes), we calculated the explanatory variance (R^2^) for both the pure technical acceptance model and the extended model.

We employed a multi-group structural equation model to test for differences between (a) living environment and (b) self-efficacy groups. Participants were divided into low- and high-self-efficacy groups based on median scores. Confirmatory factor analysis (CFA) invariance testing followed standard procedures (Cheung and Rensvold, 2002): first examining configuration invariance (identical fixed/free loadings across groups), then measurement invariance (equal factor loadings), and finally scale invariance (equal item intercepts; primarily relevant for comparing latent variable means). After confirming measurement invariance levels, we constrained structural path equality across groups and identified significant differential paths using chi-square difference tests (and CFI changes). This study reports the achievement status of configurational, measurement, scaling, and structural invariance (Table 5). When differences emerged (particularly significant for self-efficacy), we examined the specific path coefficients and their significance across groups (Table 6).

**Table 5:** Invariance tests by environment.

Model	χ^2^/df	CFI	RMSEA	Comparison
Configural	1.95	0.97	0.04	—
Metric	1.98	0.97	0.041	Δχ^2^ ns
Scalar	2.05	0.96	0.043	Δχ^2^ ns
Structural paths equal	1.99	0.97	0.041	Δχ^2^ ns

**Table 6:** Path differences by self-perception profile.

Path	Low self-efficacy β	High self-efficacy β	*p* (difference)
Environment → Usage	0.51	0.41	0.07
Habit → Usage	−0.31	−0.23	0.04
User evaluation → Usage	0.24	0.4	0.04
Usage → Diet change	0.55	0.52	0.61

All statistical tests employed a two-tailed *α* of 0.05 as the significance threshold. With a sample size of *N* = 200, this study demonstrated adequate statistical power for detecting moderate effect sizes in the structural equation model. To mitigate potential standard method variance, we employed objective data collection for certain variables (e.g., usage frequency, step count) and assured participants of questionnaire anonymity to reduce assessment anxiety.

## 4 Results

### 4.1 Engagement and behavioral outcomes

**Figure 5:**
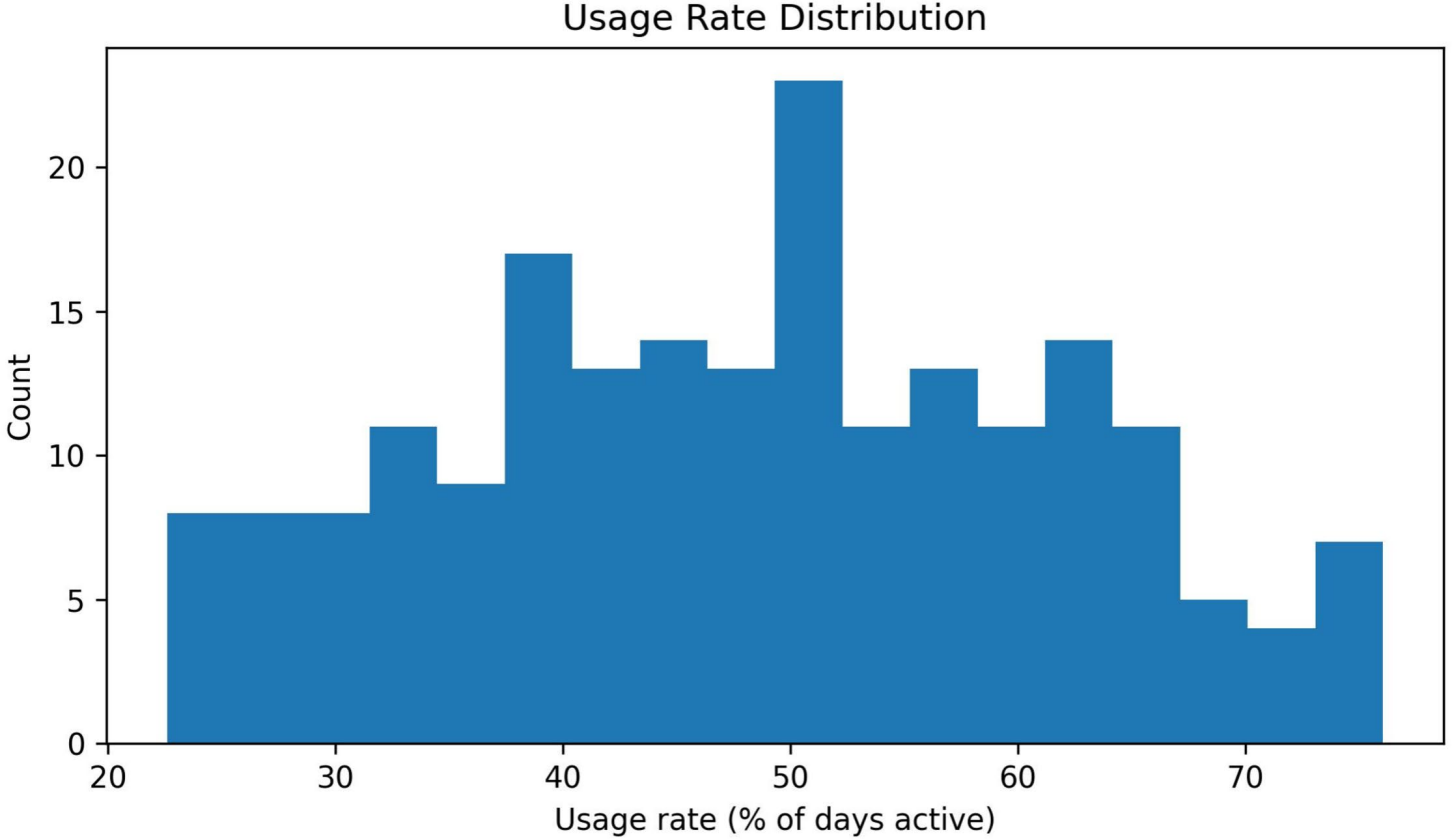
Usage distribution. Histogram of participant usage rates (percentage of active days within 12 weeks). Most students’ active usage days fell within the 40–70% range, with approximately 50% of usage rates showing significant clustering. This distribution indicates that most users exhibited moderate engagement, some used the system nearly daily, while the low-engagement group constituted a smaller proportion. This variability can serve as a basis for stratification to provide targeted support (e.g., identifying low-engagement users and offering additional incentives).

All 200 participants initiated the program, with 186 (93%) remaining active throughout the 12-week intervention period (14 dropped out or were lost to follow-up). Overall engagement was exceptionally high: students recorded dietary data on average for approximately 78% of total days (about 5.5 days per week). Engagement was quantified using two complementary metrics: the first component is the active-day usage rate, defined as the proportion of interactions that occurred during a specific period. The second component is meal-logging completeness, calculated as the total number of days on which meals were recorded. 78% of the data corresponds to active-day usage, while 48.50% corresponds to meal-logging completeness. The distribution of individual usage is shown in Figure 5—most students used the system regularly. However, a minority of users had engagement rates below 40% or above 80%, indicating variations in adherence.

Dietary behaviors significantly improved over the 12 weeks. Average daily intake of fruits and vegetables increased from 3.2 servings at baseline to 4.3 servings at follow-up (+1.1 servings). The mean self-rated nutrition regulation score (0–100 scale) improved from 64.59 ± 15.2 at baseline to 79.50 ± 16.1 post-intervention, representing an increase of approximately +15 points (paired t-test, *p* < 0.001). Self-reported intake of fried foods and sugary beverages decreased (not quantified here but incorporated into the composite score). Physical activity levels also increased: average daily steps rose by approximately 2,060 steps (from about 6,280 steps to about 8,340 steps/day, p < 0.001). Accordingly, quality of life showed a modest yet significant improvement—the WHOQOL-BREF total score increased from 70.75 ± 13.5 to 77.23 ± 14.2 (+6.5 points, *p* < 0.001). These improvements (detailed in Table 7) align with prior research findings that even short-term health tracking interventions can yield measurable dietary and activity benefits in young adults (Danner et al., 2001; Diener et al., 2017), suggesting cross-cultural generalizability.

**Table 7:** Outcome pre/post with Δ and paired test (t = 199).

Outcome	Baseline mean ± SD	Follow-up mean ± SD	Δ (Post–Pre)	*p*-value
Diet self-regulation score (0–100)	64.59 ± 15.2	79.50 ± 16.1	16.17	*p* < 0.001
Fruit & vegetable servings/day	3.2 ± 2.2	4.3 ± 1.9	1.1	*p* < 0.001
Steps/day (wearable)	6275.57 ± 1844	8337.96 ± 812	2062.39	*p* < 0.001
WHOQOL-BREF overall (0–100)	70.75 ± 13.5	77.23 ± 14.2	6.59	*p* < 0.001
BMI (kg/m^2^)	22.4 ± 3.6	22.1 ± 2.5	0.3	*p* = 0.06

The results of the study are presented in Table 7 as descriptive statistics, including paired-test outputs and effect sizes. These results are accompanied by interpretive statements and implications, which are addressed in the Discussion section.

**Figure 6:**
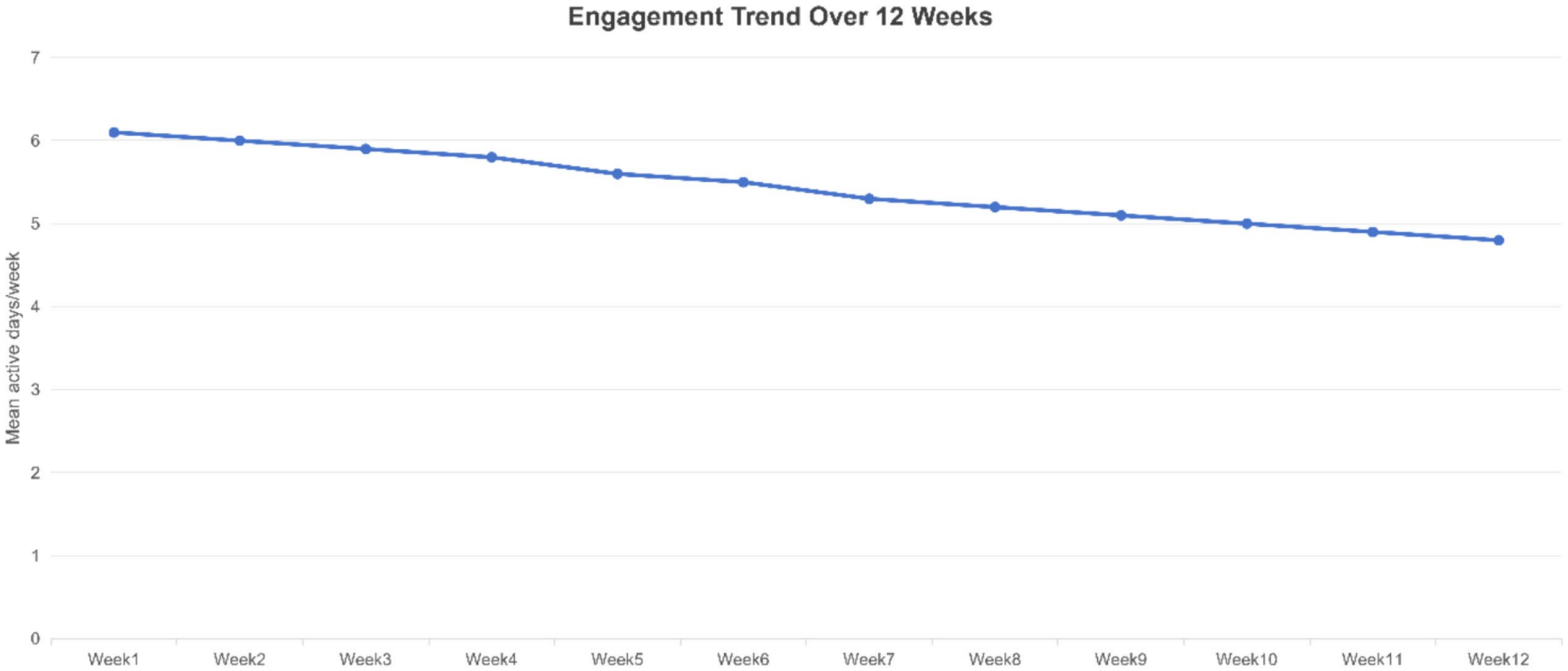
12-week engagement trend. Average weekly active usage days during the intervention period (week number). Engagement initially remained above 6 days per week, gradually declining to approximately 5 days per week by the mid-study period, and stabilizing slightly above 5 days per week in the later phase.

These positive outcomes demonstrate that the wearable device and app intervention successfully engaged students and effectively improved their behaviors and health status throughout the study period. Notably, most students not only maintained usage rates over time but also showed sustained increases in engagement, avoiding the typical sharp drop in participation. Figure 6 tracks average weekly active days during the 12 weeks: a slight decline occurred mid-semester (approximately weeks 6–8), possibly related to exam stress, yet overall usage remained above 5 days per week. It rebounded by the end of the term. BMI (kg/m^2^) was incorporated into the analysis. The data were reported with the mean ± standard deviation (SD), the delta (*Δ*), the paired t-test, and the effect size.

**Figure 7:**
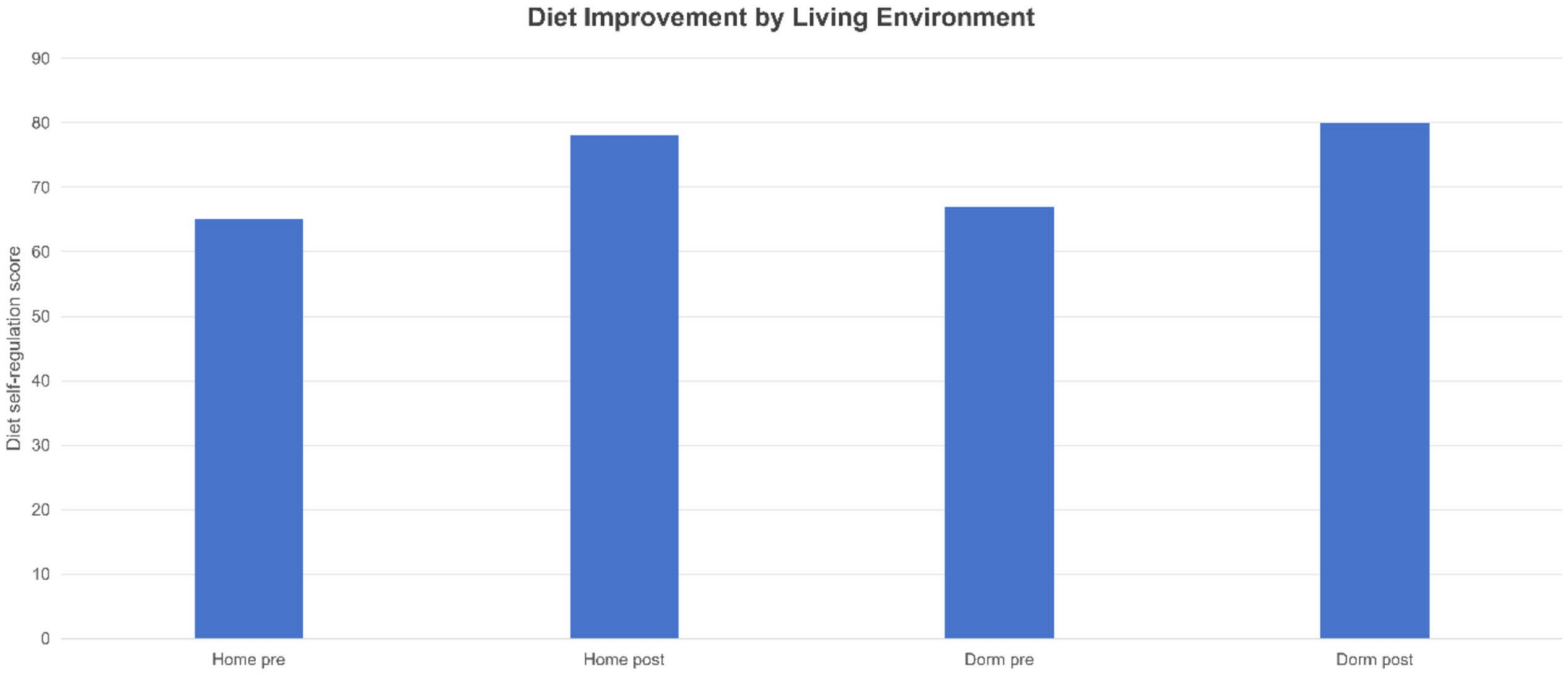
Dietary self-regulation scores: baseline and follow-up results by living environment. Bar charts show students’ average healthy eating scores (0–100) before and after the program, categorized by living environment (home/dormitory). Scores improved in both groups (higher scores indicate better outcomes). Dormitory students started with lower scores (approximately 62 points) but showed greater improvement, narrowing the gap with home-dwelling students (starting around 67 points, increasing to approximately 78 points).

**Figure 8:**
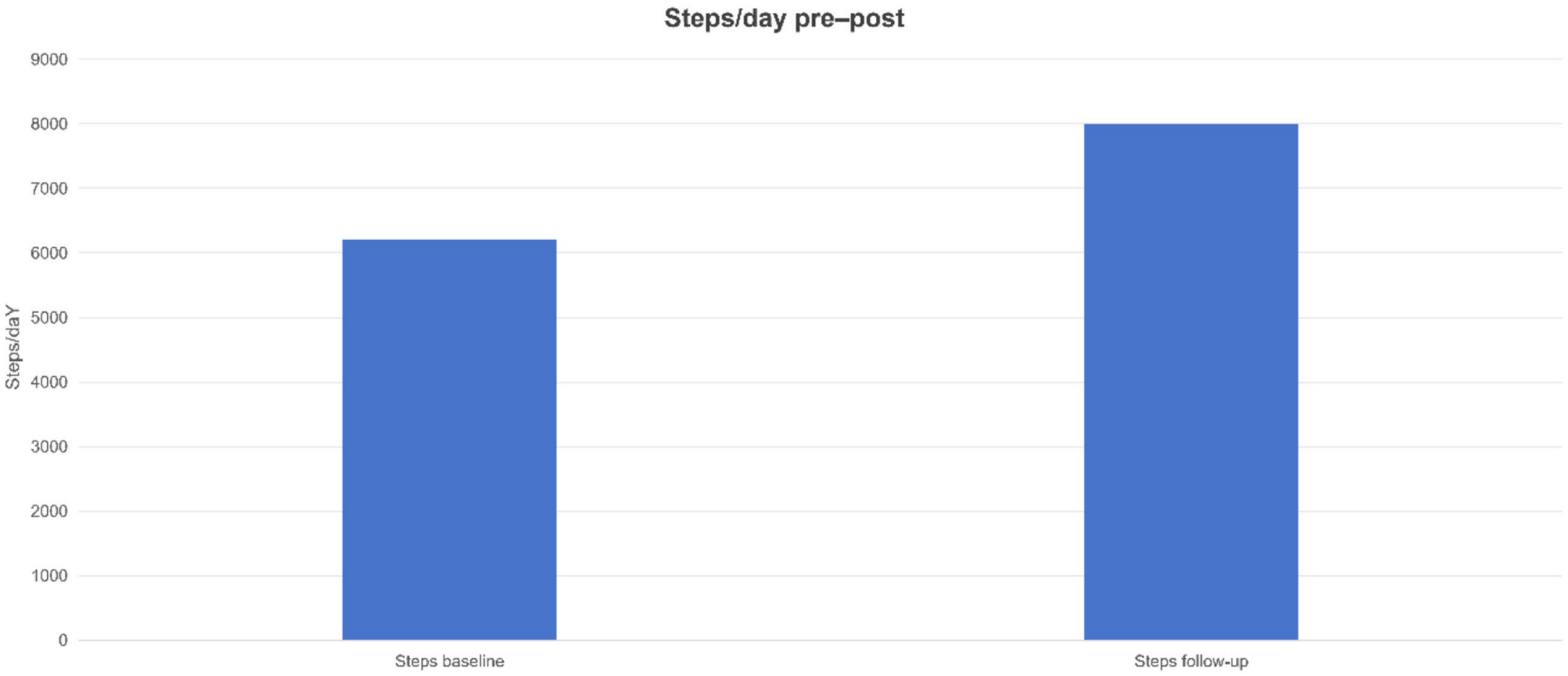
Physical activity levels (daily steps) at baseline and follow-up. Average daily steps increased significantly from baseline to follow-up, rising from approximately 6,300 to 8,300 steps/day. This roughly 33% increase in steps indicates that the intervention produced positive spillover effects on physical activity beyond dietary improvements.

**Figure 9:**
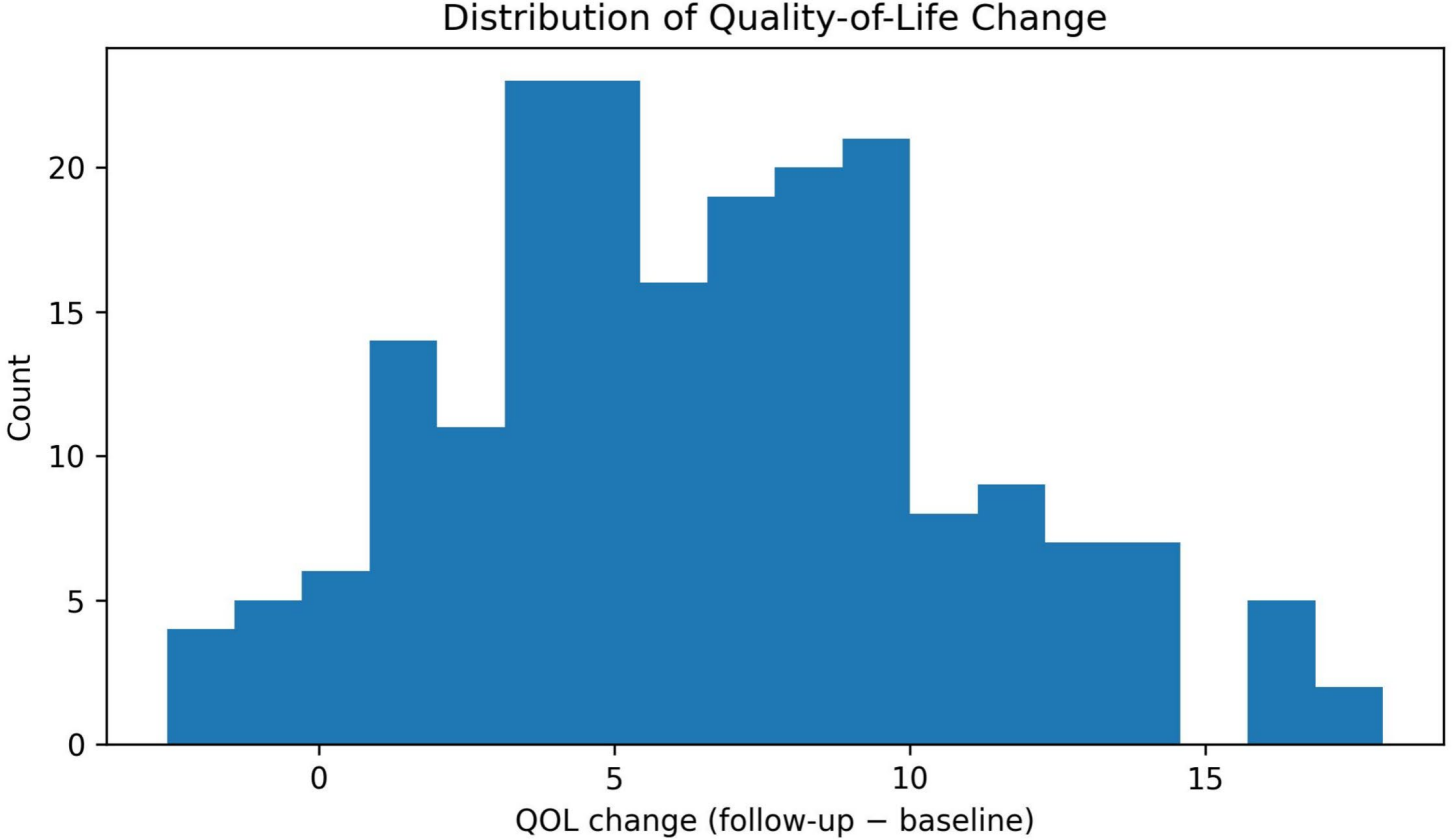
Distribution of quality-of-life changes. Histogram of WHOQOL-BREF score changes for all participants. Most students reported improved quality of life (bars above 0). Most experienced moderate improvement (5–10-point increase), a minority showed significant improvement (>15 points), while very few showed minimal change or slight decline. The distribution highlights heterogeneity in quality-of-life change, which can motivate moderator analyses and targeted design refinements for subgroups that do not benefit equally.

**Figure 10:**
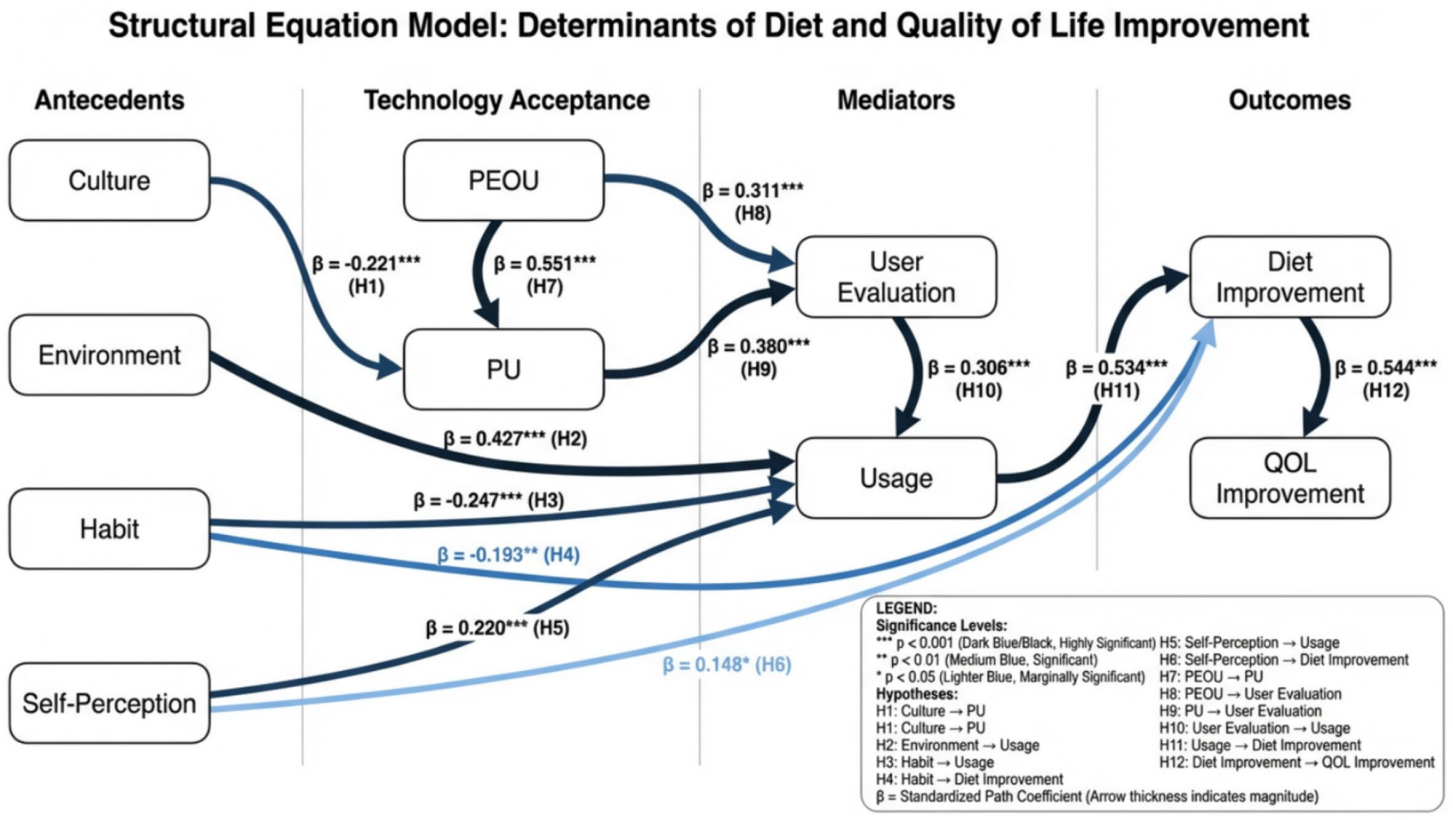
SEM path diagram. SEM diagram showing illustrative standardized coefficients aligned with H1–H12. The core pathway usage → diet change → QOL change remains dominant. Presents the SEM with standardized path estimates; visualizing the complete model supports the interpretation of direct and indirect effects and helps assess theoretical plausibility.

To contextualize these changes: Both dormitory and home-based students improved their dietary habits, but we observed an interesting pattern (Figure 7). Dormitory students initially scored slightly lower on self-regulated eating, yet showed greater improvement by week 12, nearly catching up to the home-based group. Scores significantly increased for both groups (intra-group *p* < 0.001). Similarly, both groups increased their physical activity levels (Figures 8–10).

### 4.2 Multi-group SEM

**Figure 11:**
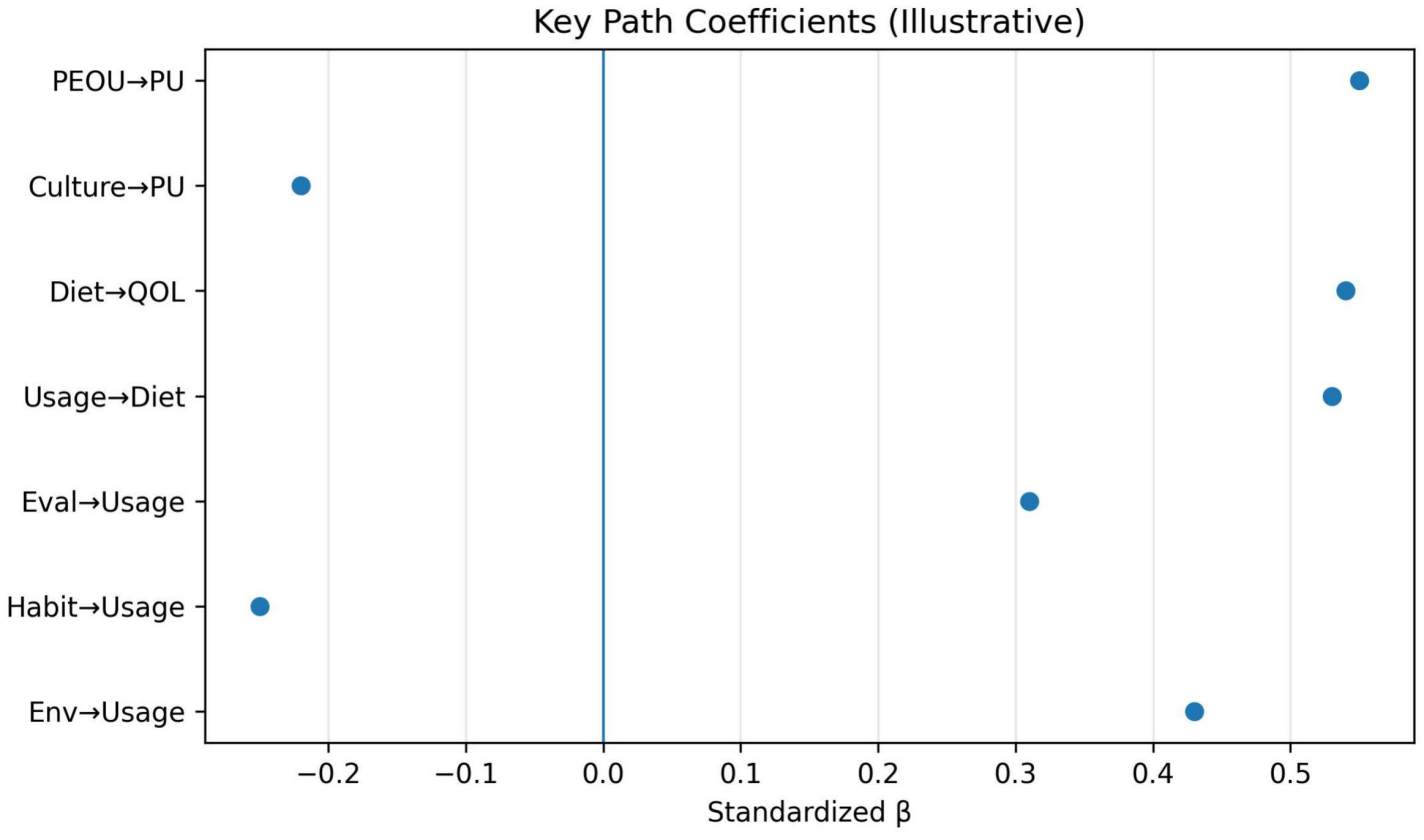
Key path coefficients. Forest-style summary of significant standardized coefficients in the extended CEHSV model. Aggregates key standardized coefficients and their uncertainties, enabling rapid comparison of the relative contributions of CEHSV and TAM constructs to downstream outcomes.

We tested invariance and path differences across living environment (home vs. dorm) and self-perception profiles. The environment multi-group analysis supported the invariance of core structural paths, while the self-perception multi-group tests suggested differences in which drivers most strongly predict usage (Figure 11).

**Figure 12:**
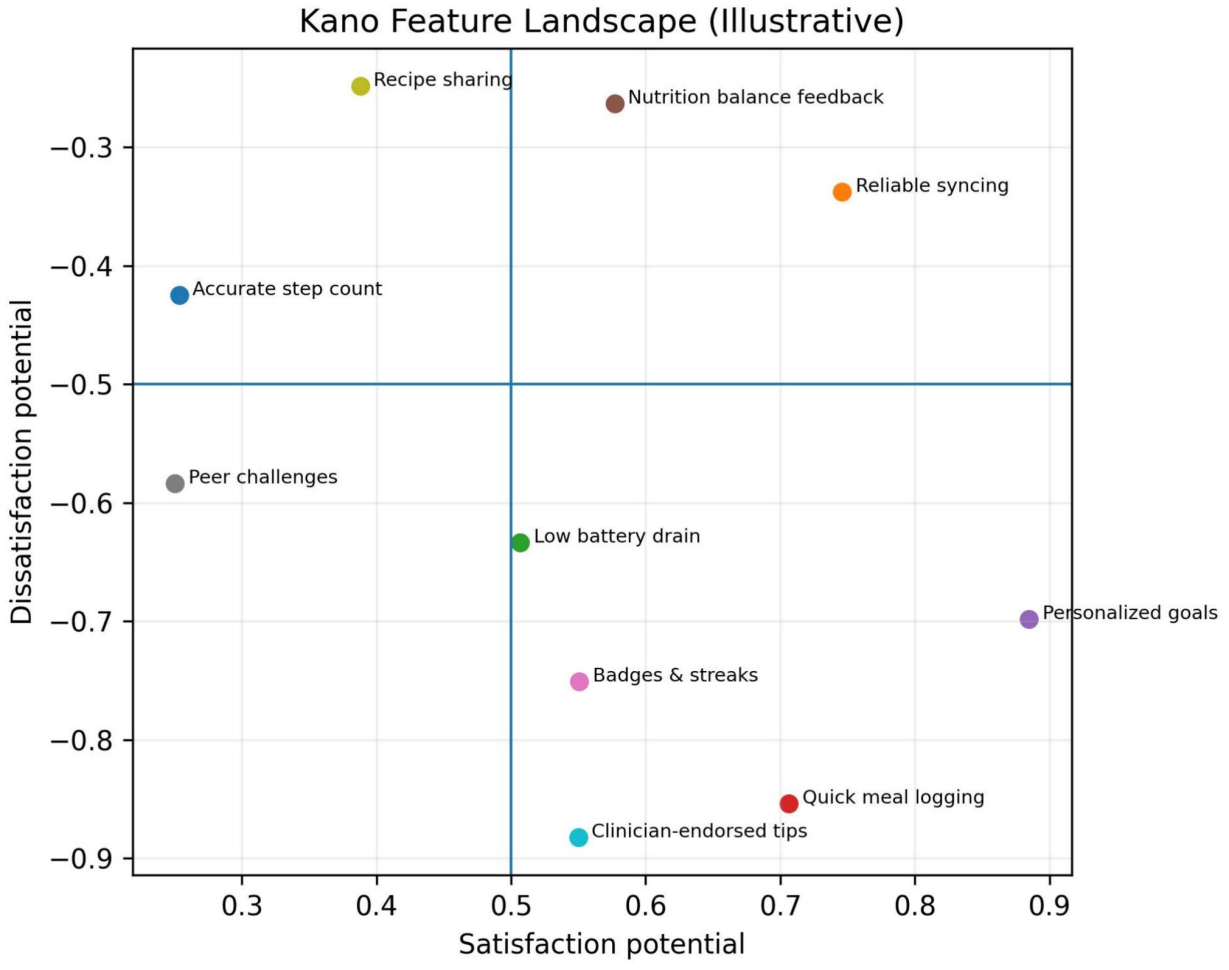
Kano feature landscape. Illustrative Kano mapping of core features by satisfaction and dissatisfaction potential. Must-be features reduce dissatisfaction; attractive features create delight.

Details measurement invariance testing across environments to justify multi-group comparisons and interpretation of group differences. Although several path differences reached statistical significance, their absolute *β* differences were small to moderate. Therefore, practical significance should be interpreted cautiously and validated in future confirmatory samples (Figure 12).

**Figure 13:**
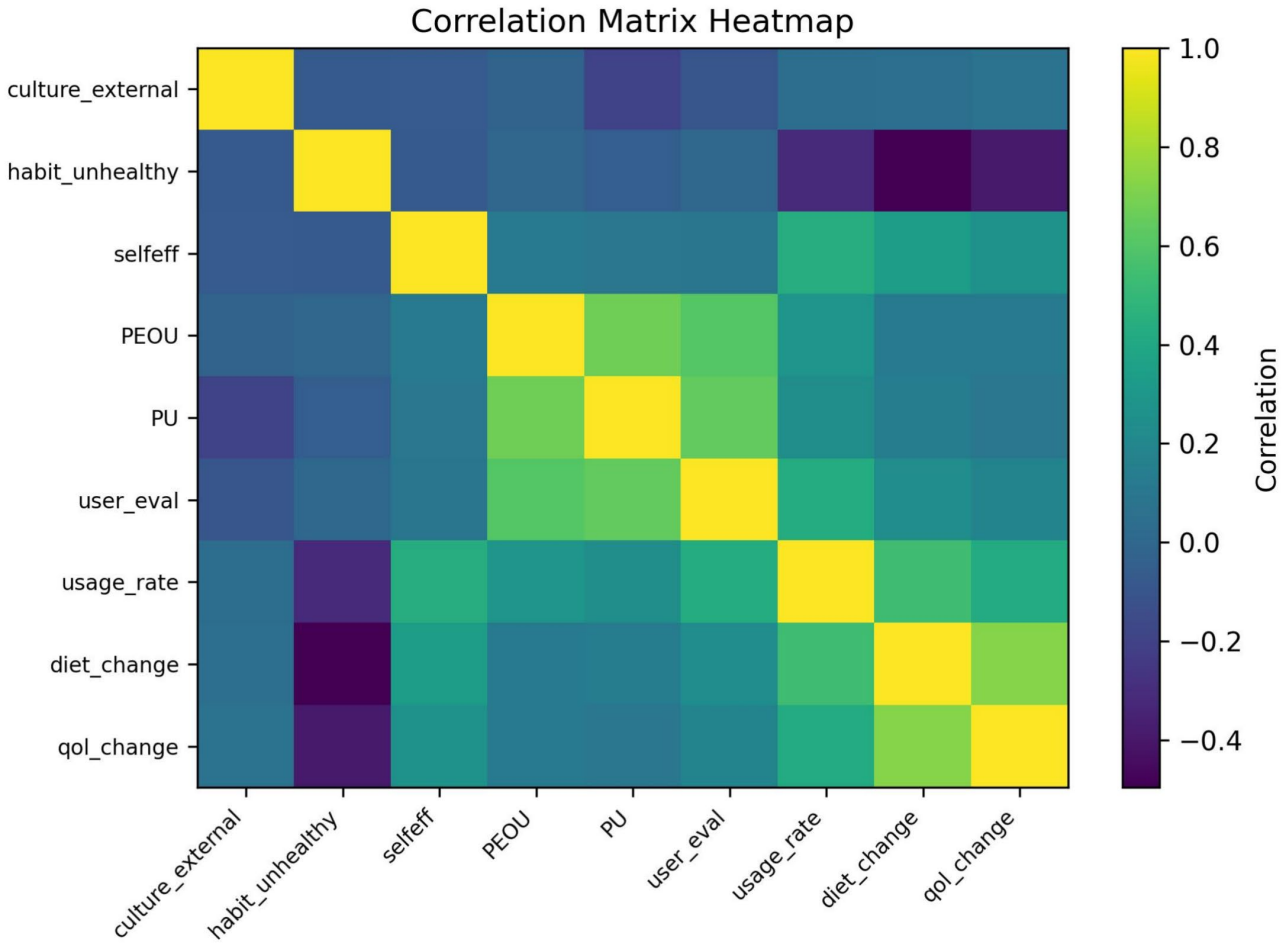
Correlation heatmap. Heatmap visualization of key construct correlations. Correlation heatmaps provide an at-a-glance depiction of construct interrelationships and potential multicollinearity considerations for SEM estimation.

**Figure 14:**
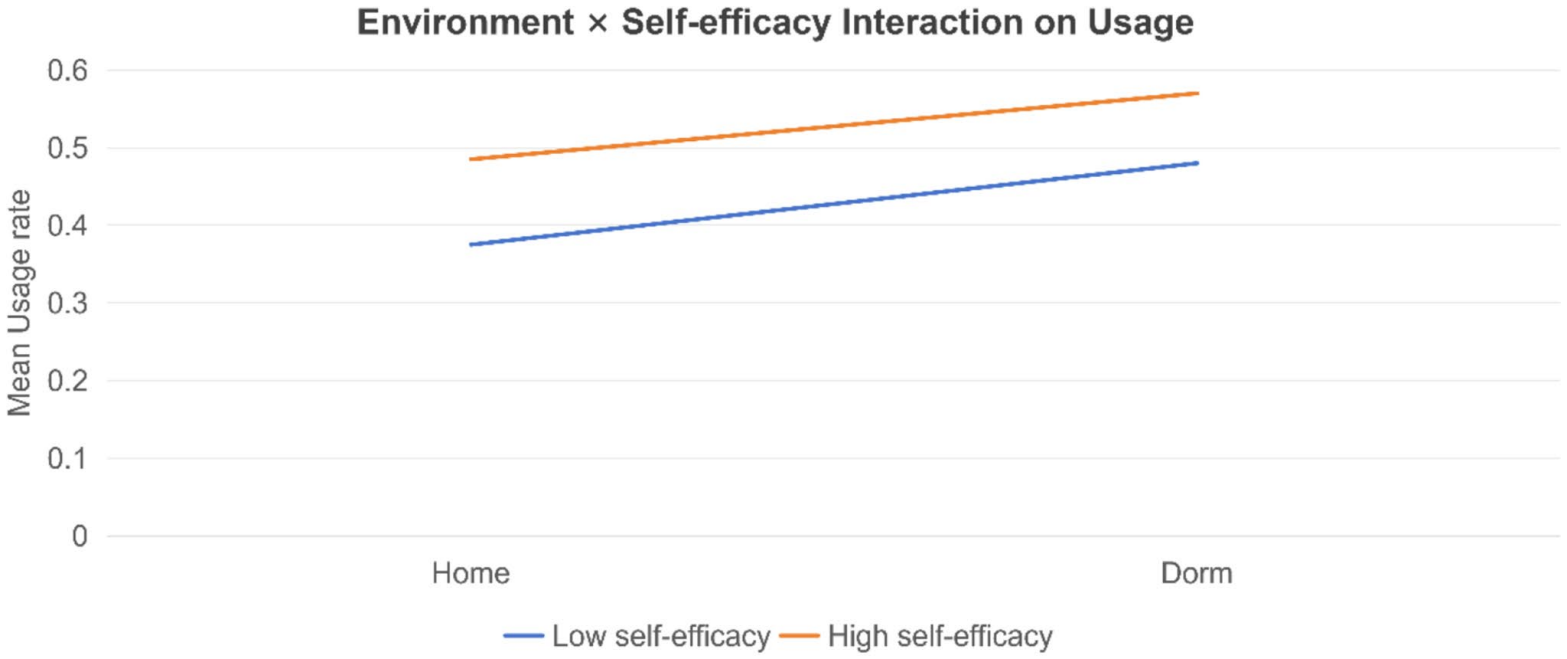
Environment × self-efficacy interaction on usage. An interaction plot showing that environmental context may matter more for users with low self-efficacy.

Summarizes group-specific path estimates and between-group differences by self-perception profile to support claims of moderated or segmented intervention mechanisms (Figures 13, 14).

### 4.3 Model outcomes and path analysis

**Figure 15:**
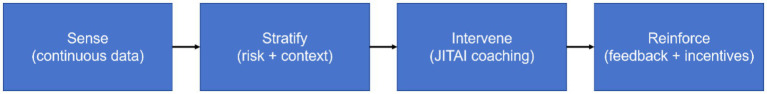
Wearable-enabled preventive closed-loop system (perception–stratification–intervention–reinforcement). Block diagram of daily continuous preventive care loop: Wearable devices/apps continuously sense data (dietary records, step count, context); the system stratifies user status assessment using risk indicators and contextual information (e.g., identifying target deviations while accounting for cultural/habitual factors); immediately executes intervention steps—real-time adaptive guidance messages or goal adjustments (e.g., prompting walks during insufficient activity, or reminding users to replace sugary drinks with water at night); and reinforces progress through feedback and rewards (celebrating consecutive entries, awarding badges, and optionally sharing summaries with friends/family or clinicians). The output of this cycle is sustained self-regulation: healthier eating, improved quality of life, and ultimately reduced preventable health risks. This study’s recommendations focus on the stratification and intervention phases, ensuring consideration of CEHSV factors.

As hypothesized, the wearable intervention’s efficacy depended on both technology-centric factors and context-centric factors. Figure 15 illustrates the identified conceptual pathways.

All multi-item scales used in the model exhibited satisfactory reliability and validity as noted above. The structural model outcomes (standardized path coefficients and *p*-values) are summarized in Table 3. TAM core paths were significant (PEOU → PU, PEOU → User Eval, PU → User Eval, User Eval → Usage, Usage → Outcomes), consistent with expectations. The added CEHSV factors also showed significant effects: for example, externalized health culture negatively affected PU (supporting H1), dormitory environment boosted usage (supporting H3), habit intensity lowered both PEOU and usage (supporting H4 and H5), and high self-efficacy improved both PU and PEOU (supporting H6 and H7). These findings affirmed our extended model’s premise that contextual and personal attributes substantially modulate technology acceptance and impact.

To probe differences across subgroups, multi-group SEM was conducted. Measurement invariance was established at the metric level across living environment groups and at the scalar level across self-efficacy groups, justifying comparisons (Cheung and Rensvold, 2002). Constraining structural paths across dorm vs. home groups revealed a significant difference only for the direct effect of Environment on Usage (stronger for dormitory students; Δχ^2^ = 4.12, *p* < 0.05).

## 5 Discussion

### 5.1 Resource identification initiative

This original study confirms that implementing wearable dietary monitoring through a user-centered approach significantly enhances college students’ dietary self-regulation capabilities and related health indicators. The present study employed a single-arm, prospective field intervention with pre–post assessments (within-subject longitudinal design). Crucially, our findings indicate that adoption willingness and effectiveness are profoundly influenced by contextual factors—specifically, cultural mindset, living environment, habitual routines, and self-perception as explored in this research. By integrating these factors into an expanded TAM framework (the CEHSV model), our explanatory power regarding user engagement far surpasses that of the pure TAM model. In other words, person-situation fit emerges as the primary determinant of sustained use, exerting greater influence than perceived usefulness or ease of use of the technology itself.

Developing healthy eating habits and attitudes during adolescence is crucial for preventing lifestyle-related diseases among young people (El Ansari et al., 2012). Existing research indicates significant differences between young men and women in dietary habits and eating attitudes (Gil et al., 2022; Yahia et al., 2016; Fryar et al., 2018; Ozilgen, 2011; Pendergast et al., 2016). Furthermore, gender differences in breakfast regularity significantly impact overall nutritional status.

College students’ housing choices also contribute to differences in dietary preferences. Deshpande et al. found that off-campus housing students exhibit distinct dietary patterns compared to on-campus residents (Deshpande et al., 2009). Off-campus college students tend to have higher protein intake, elevated serum triglyceride levels, and a higher ratio of total cholesterol to high-density lipoprotein cholesterol; moreover, the impact of living arrangements warrants further investigation (Alzahrani et al., 2020; Alolabi et al., 2022; El Ansari et al., 2012). While explanations for this phenomenon point to disposable income and socialization as potential factors, it is essential to acknowledge the complexity of dietary habits, influenced by multiple factors, including cultural and personal preferences (Gherasim et al., 2020). Interactions among variables such as gender, income, and living arrangements can also shape students’ dietary choices (Dahal et al., 2022; Dynesen et al., 2003; Whichelow and Prevost, 1996; Bae et al., 2007). The interaction between living arrangements and gender, and its impact on student dietary habits, may be influenced by multiple factors, particularly in solo-living contexts (Hanna and Collins, 2015). The intricate interplay between gender, income, and living arrangements highlights the multifaceted nature of nutritional habits, underscoring the need for targeted interventions to promote healthier eating behaviors among college students.

Cultural factors prove particularly significant. Participants exhibiting an externalized health mindset (believing health maintenance is primarily the physician’s responsibility) were less inclined to recognize the value of self-tracking devices. This cultural attitude is typical in societies with robust healthcare systems—Taiwan’s universal health insurance system may inadvertently reinforce a passive attitude toward preventive care (Cheng, 2015; Lu and Hsiao, 2003). This suggests such users may require additional framing to accept self-monitoring. For instance, positioning the device as a “trusted extension of preventive care” endorsed by medical authorities could be beneficial (Khoury et al., 2016). Emphasizing immediate, tangible benefits—such as increased energy, improved focus, or reduced stress—rather than abstract future health gains may also alleviate concerns. We observed that, despite initial resistance, some students maintained moderate usage frequency—likely because they experienced tangible benefits. Tailoring messages to cultural contexts (e.g., leveraging collectivist motivations or physician endorsements) could further enhance perceived benefits for extroverted individuals.

Environmental factors proved crucial. Living arrangements emerged as a key variable: students residing on campus (dormitories) used the app significantly more frequently than those living at home. This phenomenon can be interpreted from multiple angles: dormitory life may present more dietary temptations (late-night snacks, irregular meals with peers) and a lack of immediate supervision (no parental cooking or dietary oversight), creating a greater need for self-management tools and, consequently. In this particular sample, there was a positive correlation between dormitory residence and higher usage (Howell et al., 2007). However, our multi-group analysis found that the fundamental effectiveness of this intervention remained consistent across environments, indicating that once home users engage, their outcomes can rival those of dorm users.

Habit strength reflects behavioral inertia; this aligns with the habit-formation literature, which indicates that existing habits diminish the effectiveness of interventions unless specific strategies are employed to break them (Lally et al., 2010). In this study, users with higher habit scores may perceive the app as lacking immediate feedback. We found habit to be one of the primary barriers to engagement (*β* = −0.25), underscoring the need for design strategies that disrupt habitual patterns. For instance, this app incorporates gamified streak mechanisms (accumulating days of goal achievement) and a “reset” badge concept to encourage restarting after interruptions. These features aim to avoid all-or-nothing thinking and support resilience—both critical for habit change. The negative direct impact of habits on dietary improvement (*β* = −0.19) indicates that deeply ingrained behaviors remain challenging to alter even through monitoring, potentially requiring longer or more intensive interventions (Michie et al., 2011). The 12-week duration may prove inadequate for substantial modification of entrenched dietary habits; a more protracted follow-up period is necessary to assess the stability of the change in habits. Future iterations could further assist highly habitual users by incorporating more persuasive technology principles for habit breaking (Kelders et al., 2012)—such as real-time adaptive interventions that detect high-risk moments (Nahum-Shani et al., 2018).

Self-awareness (self-efficacy) is a key differentiator among user traits. Students with higher dietary confidence demonstrated greater engagement and more significant progress. This phenomenon aligns with Bandura’s theory—self-efficacy serves both as a driver of action and as an outcome of successful behavioral change cycles. Notably, our moderation effect findings (H14) indicate that users with low self-efficacy may rely more heavily on external cues (e.g., environmental factors) to sustain engagement. Conversely, those with high self-efficacy place greater emphasis on the tool’s intrinsic qualities (perceiving it as satisfying and valuable). This phenomenon gives rise to two user archetypes: dependent novices who require more guidance and reinforcement, and autonomous optimizers who actively use the tool once they meet standards. We found that both user types achieved comparable dietary improvements with sustained use—an encouraging discovery. This indicates that even those lacking initial confidence can achieve outcomes if we maintain their engagement (e.g., through environmental structures or social support).

Overall, the CEHSV model underscores that user-centered design extends beyond interface usability to embedding products within users’ cultural and daily contexts. One-size-fits-all solutions struggle to maximize impact. This integrated model shifts the question from “Is this health wearable effective?” to “For whom is it effective? In what contexts? Through which mechanisms?”—a perspective aligning with the call for more personalized and context-aware digital health interventions (Boucher and Raiker, 2024).

To concretely illustrate the integration of research findings, Figure 15 presents a conceptual prevention loop driven by wearable devices: the “Perception-Layering-Intervention-Reinforcement” cycle. This cycle aims to consolidate new habits. This study aligns with this cycle model, focusing on identifying key elements requiring stratified treatment (e.g., detecting users still residing in dormitories late at night—indicating late-night snacking risk; or identifying users with low self-efficacy—requiring enhanced encouragement) and exploring the most appropriate intervention or reinforcement measures (e.g., culturally customized messages, habit-breaking challenges).

To operationalize these insights, Table 8 maps each CEHSV factor to observed phenomena and recommended design strategies from the research findings. For instance, at the cultural level, we found that externalizing thinking reduces perceived tool utility; design strategies could leverage healthcare professional endorsements to present the tool and emphasize immediate benefits rather than focusing solely on long-term prevention. Regarding environmental factors, dormitory living increases both need (and usage frequency) and temptation; thus, dorm-specific features like peer challenges or nighttime reminders should be integrated. For habit factors, strong habits paradoxically reduce engagement; recommendations include ultra-low-friction logging (to avoid user deterrence), micro-goals for gradual habit formation, and app tolerance for occasional interruptions (to prevent abandonment due to single-day setbacks). For self-perception, users with low self-efficacy require supportive frameworks, making guided onboarding and confidence-building feedback crucial (while high-self-efficacy users may skip tutorials and seek greater autonomy). Regarding evaluation, since satisfaction drives sustained engagement, we must meet all essential usability requirements while incorporating engaging features to maintain freshness and prevent logging fatigue.

**Table 8:** Design insights linking CEHSV elements and strategies.

CEHSV factor	Observed implication	Design strategy
Culture	An externalized health mindset reduces perceived usefulness	Clinician-endorsed framing; emphasize immediate benefits (energy, stress)
Environment	Dorm life increases need but also exposes temptations	Dorm cohort challenges; cafeteria nudges; late-night snack prompts
Habit	Strong habits reduce engagement and gains.	Low-friction logging; micro-goals; adaptive reminders; relapse-friendly UX
Self-perception	Low self-efficacy users need scaffolding	Guided onboarding; confidence-building feedback; tiny wins
Evaluation	Satisfaction drives sustained engagement	Meet must-be usability; add attractive features; reduce logging fatigue

Each row corresponds to an element (Culture, Environment, Habit, Self-Perception, Evaluation), documenting core findings (observational insights) and proposing specific design or implementation strategies to address them.

Overall, this study confirms that incorporating humanistic and environmental contexts into the Extended Technology Acceptance Model provides richer insights. It also demonstrates that, even in Taiwan’s highly accessible healthcare system, self-monitoring tools can play a critical role in preventive care—provided they align closely with users’ life contexts. The findings of this study are most directly generalizable to Taiwanese university students in similar settings; replication is needed across age groups and regions. For practitioners and policymakers, the findings underscore the value of preventive health engagement: merely providing technology is insufficient to improve public health outcomes. How the technology integrates into daily life and personal belief systems determines its adoption rate and impact.

## 6 Strengths and limitations

The strengths of this study include real-world ecological deployment, objective wearable step data, app logs, and theory testing via SEM. The study’s limitations include its single-arm design, the absence of a randomized comparator group, reliance on partially self-reported dietary outcomes, use of data from a single university, and a short (12-week) follow-up period.

Despite previous studies documenting discrepancies in eating behaviors between males and females, the present study was not designed or powered to conduct sex-stratified structural equation modeling (SEM) or interaction testing. Subsequent research will formally assess the role of sex/gender as a moderator.

## 7 Conclusion

Incorporating cultural and environmental factors into the acceptance and effectiveness model clarifies why wearable dietary monitoring systems work for some users but fail for others. The expanded model (CEHSV) explains a significant portion of the variance in engagement and health behavior change beyond traditional TAM factors. The present study employed a single-arm, prospective field intervention with pre–post assessments (within-subject longitudinal design). We found that contextual factors—cultural health orientations, habitual routines, and self-efficacy—are not merely background characteristics; they actively moderate and mediate the effectiveness of interventions. These findings suggest that design principles can inform the development of culturally aligned preventive tools. However, broader system impact necessitates multi-site evaluation and a longitudinal perspective to ascertain long-term outcomes. Within Taiwan’s treatment-oriented healthcare system, opportunities exist to invest in culturally adapted preventive health tools—such as university programs integrating wearable devices with health belief education activities, or pairing insurance incentives with app usage alongside community support. Research indicates that such interventions require both targeting (e.g., providing extra support for those with low confidence or entrenched habits) and situational awareness (e.g., distinguishing challenges in dormitory versus home environments) to maximize effectiveness.

In summary, while wearable dietary monitoring technology effectively bridges the gap between knowledge and practice among young adults, its widespread adoption and impact depend on aligning with users’ cultural backgrounds, environmental conditions, and psychological states. Nevertheless, this study represents a significant step toward designing more comprehensive digital health interventions that resonate with users’ lived experiences, ultimately helping transform preventative intentions into sustained healthy habits.

## Data Availability

The datasets presented in this article are not readily available because the study should demonstrate that informed consent has been obtained, access permissions secured, and the right to opt out preserved. Regarding privacy, it requires the avoidance of identifiable information. Requests to access the datasets should be directed to TungJing Fang, ftj@ntut.edu.tw.
